# Normalization of photoplethysmography using deep neural networks for individual and group comparison

**DOI:** 10.1038/s41598-022-07107-5

**Published:** 2022-02-24

**Authors:** Ji Woon Kim, Seong-Wook Choi

**Affiliations:** 1 Interdisciplinary Program in Biohealth-Machinery Convergence Engineering, Kangwon National University, Chuncheon-si, 24341 Korea; 2 Program of Mechanical and Biomedical Engineering, College of Engineering, Chuncheon-si, 24341 Korea

**Keywords:** Health care, Engineering

## Abstract

Photoplethysmography (PPG) is easy to measure and provides important parameters related to heart rate and arrhythmia. However, automated PPG methods have not been developed because of their susceptibility to motion artifacts and differences in waveform characteristics among individuals. With increasing use of telemedicine, there is growing interest in application of deep neural network (DNN) technology for efficient analysis of vast amounts of PPG data. This study is about an algorithm for measuring a patient's PPG and comparing it with their own data stored previously and with the average data of several groups. Six deep neural networks were used to normalize the PPG waveform according to the heart rate by removing uninformative regions from the PPG, distinguishing between heartbeat and reflection pulses, dividing the heartbeat waveform into 10 segments and averaging the values according to each segments. PPG data were measured using telemedicine in both groups. Group 1 consisted of healthy people aged 25 to 35 years, and Group 2 consisted of patients between 60 and 75 years of age taking antihypertensive medications. The proposed algorithm could accurately determine which group the subject belonged with the newly measured PPG data (AUC = 0.998). On the other hand, errors were frequently observed in identification of individuals (AUC = 0.819).

## Introduction

As the demand for noncontact telemedicine services increases, the amount of medical data directly measured by patients themselves outside the hospital setting is increasing^1^. To improve the reliability of remotely transmitted medical data, a technology capable of capturing the characteristics of individuals and patients with diseases is required^2^. Photoplethysmography (PPG) is one of the most frequently used medical monitoring technologies due its convenience, and it has been employed to predict the likelihood of disease and for identification of individuals^3–8^. However, as most conventional automatic PPG analyzers have difficulty in distinguishing waveforms caused by motion artifacts and electrode-connection failures, etc., to increase the reliability of automatic analysis requires deep neural network (DNN) technology for normalization of PPG waveforms and to exclude artifacts^9–11^.

Although many methods for determining the likelihood of disease and identifying individual characteristics have been proposed, they have the limitation of requiring human experts to determine whether the PPG waves are informative or uninformative^5–8^. Also, these methods have not been validated because it is difficult to obtain sufficient data through manual analysis. Therefore, a number of DNN techniques have been applied with the goal of replacing human experts in PPG analysis. However, their accuracy remains low and more training data are required in the machine learning process to increase the accuracy of these DNNs^12–19^.

Recently, telemedicine in Korea has generated large amounts of PPG data^20^. DNN models have been developed to select reliable data from the transmitted PPGs and normalize the PPG waveform according to the heart-rate (HR) cycle, while excluding data affected by motion artifacts or electrode connection failures^21–24^. Even if PPG data were measured by the same person, the waveform of PPG continuously changes due to patient stress or unknown causes, so it is necessary to automatically measure as much high-quality data as possible using DNNs. It is expected that a novel DNN algorithm for automatically normalizing the many PPG data will improve the accuracy of PPG analysis for individual and group identification.

The purpose of this study is to show that, even though the PPG data changes daily and includes a lot of noise, the differences of individuals and groups can be identified by the suggested method. The six DNN models in this study identify features of the heartbeat in PPG waveforms and evaluate whether the PPG waves are informative. The algorithm selects the informative region in the PPG and divides it into 10 sections according to the phases of the HR cycle. Then, the mean ± standard deviation of each section is calculated for use as criteria in individual and two-group identification; Group 1 consisted of healthy people aged 25 to 35 years, and Group 2 consisted of patients aged 60 and 75 years taking antihypertensive medications.

## Results

### Up personal identification using PPG data

**Figure 1:**
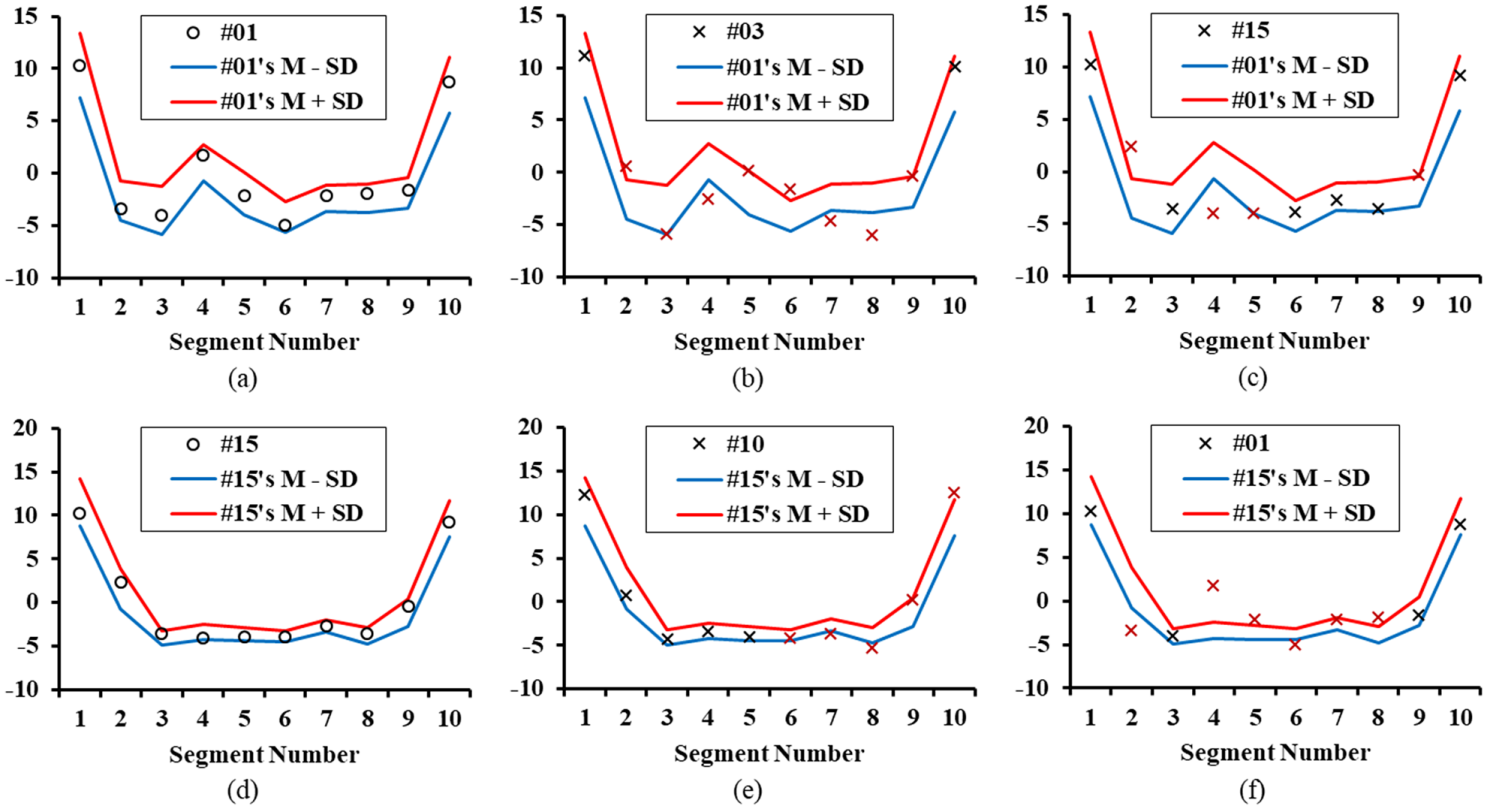
Examples of individual identification; (**a**) when the normalized data of subject #1 in Group 1 was compared with subject #1’s reference data, (**b**) when subject #3’s data in Group 1 was compared with subject #1’s reference data, (**c**) when subject #15’s data in Group 2 was compared with subject #1’s reference data, (**d**) when subject #15’s data was compared with subject #15’s reference data, (**e**) when subject #10’s data in Group 2 was compared with subject #15’s reference data, and (**f**) when subject #1’s data in Group 1 was compared with subject #15’s reference data.

Figure 1a–c show example PPG data from subjects #1, #3, and #15, respectively, for individual identification based on previously obtained section-specific data. Although subjects #1 and #3 both belonged to the Group 1, their data showed some differences, as did those of subject #1 compared to one of Group 2 (subject #15). Each individual’s statistical data were obtained during 10 sessions, and the mean PPG velocity ± standard deviation was calculated for each session in advance by analyzing the data of at least 120 heartbeats. The statistical data were obtained in five measurement periods and were normalized according to the heart rate cycle. In Fig. 1, the top (red) and bottom (blue) lines are the criteria for subject #1, and were calculated as the sum and difference of the mean and standard deviation per session, respectively. The newly measured values of subject #1 are consistent with the criteria of subject #1, shown as lines in Fig. 1a. As shown in Fig. 1b, the PPG waveform of subject #3 was similar to that of subject #1, in that the reflection wave was larger than that of Group 2. However, there was a difference in reflection wave occurrence. If this method can find differences even among individuals within the same group, it may be possible to distinguish individuals based on the waveform characteristics. However, common characteristics among subjects in Group 1 can lead to many errors in individual identification. As Group 2 shows distinct differences from Group 1, the possibility of misidentifying a subject in Group 2 as Group 1 is low.

Figure 1d–f compare the PPG data of subjects #15, #10, and #1 with the statistical data of subject #15. The newly measured PPG data of subject #15 show high consistency with the statistical data of subject #15, as shown in Fig. 1d. Figure 1e shows that, although subjects #15 and #10 were the same age and had small reflection waves on PPG in common, they were recognized as distinct individuals based on differences in their pulsation waves. However, the common features and simplicity of the waveforms among the subjects in Group 2 lead to a high probability of individual identification failure.

**Figure 2:**
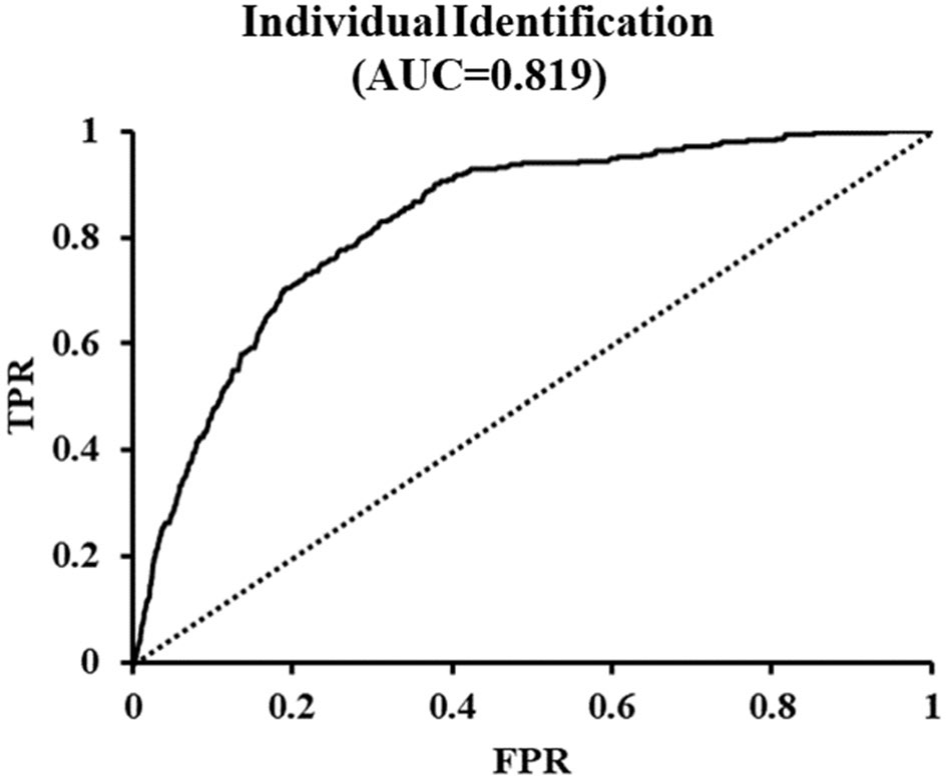
Receiver operating characteristic (ROC) curves of individual identification when new PPG data (353) measured from all subjects were compared with each individual reference data (#1 ~ #15).

The receiver operating characteristic (ROC) curve in Fig. 2 shows the sensitivity (true-positive rate, TPR) and false-positive rate (FPR), which distinguishes each individual from all other individuals. Z-values are normalized to the differences between newly measured PPG data and previous statistical data (by averaging and dividing by the standard deviation). The area under the curve (AUC) value of 0.819 indicates that identification of individuals is inaccurate, but possible to a limited extent.

### Patient group identification using PPG data

Table 1 shows the differences of beat-to-beat PPG waves between Group 1 and Group 2 in each of the 10 sessions. In order to clearly confirm whether there are differences between the two groups in the reference data measured 5 times from the first 15 people, t-values considering the number of participants were included to Table 1. Considering the number of participants (t_0.05, 7_ = 1.895), segments 4 and 5 showed a clear difference (p = 0.05). Table 1 showed that these differences of 4 and 5 segments were also observed in the results obtained from 353 newly measured data.

**Table 1:** Differences in PPG waveforms between the Group 1 and Group 2.

Segment number	Group 1 (n = 8)	Group 2 (n = 7)	Standard deviation of reference data ${\mathrm{SD}}_{\mathrm{1,2}}=\sqrt{{{{SD}_{1}}^{2}/8}} +\sqrt{{{{SD}_{2}}^{2}/7}}$	t-test of reference data (t_0.05, 7_ = 1.895)	Mean difference of test data (= *M*_*1*_*—M*_*2*_*, n* = *353*)
	Average (*M*_*1*_) ± standard deviation (*SD*_*1*_)	Average (*M*_*2*_) ± standard deviation (*SD*_*2*_)			
1	10.589 ± 1.955	10.333 ± 2.394	1.139	0.224	0.254
2	0.049 ± 1.592	1.846 ± 2.086	0.969	− 1.855	− 1.783
3	− 4.449 ± 1.802	− 3.467 ± 1.137	0.768	− 1.279	− 1.071
4	− 2.028 ± 1.692	− 3.655 ± 0.918	0.691	2.354	1.639
5	− 1.685 ± 1.386	− 3.792 ± 0.595	0.539	3.906	2.167
6	− 2.921 ± 1.390	− 3.709 ± 0.610	0.543	1.452	0.794
7	− 3.263 ± 1.227	− 2.931 ± 0.731	0.514	− 0.645	− 0.335
8	− 4.142 ± 1.514	− 3.764 ± 0.995	0.654	− 0.578	− 0.416
9	− 0.940 ± 1.420	− 0.389 ± 1.951	0.892	− 0.617	− 0.531
10	9.241 ± 2.014	9.540 ± 2.639	1.226	− 0.244	− 0.319

**Figure 3:**
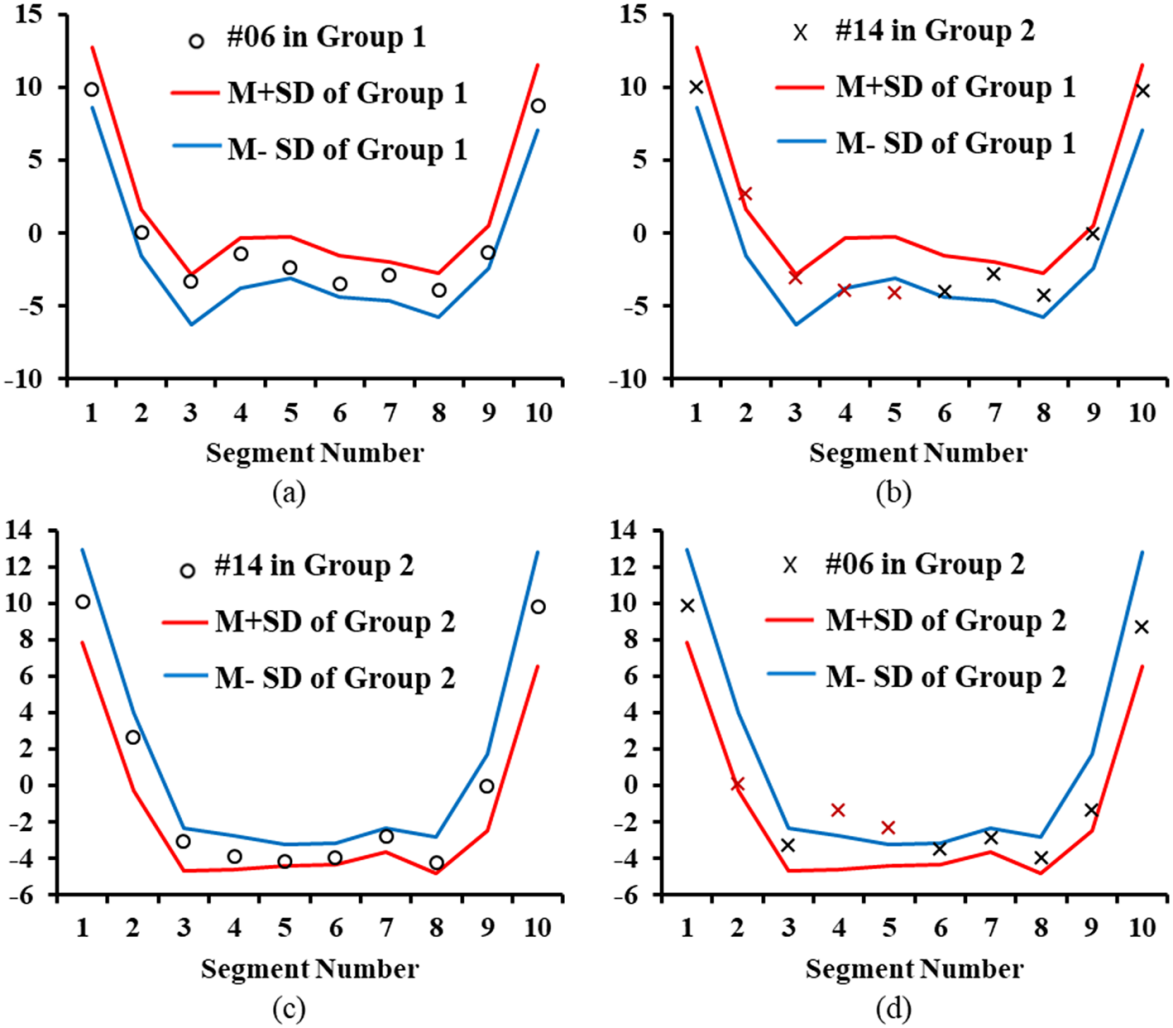
Example group identification data of subjects #6 and #14; (**a**) comparing subject #6’s normalized data in Group 1 with the reference data of Group 1, (**b**) comparing subject #14’s data in Group 2 with the reference data of Group 1, (**c**) comparing subject #14’s data with the reference data of Group 2 and (**d**) comparing subject #6’s data with the reference data of Group 2.

The lines in Fig. 3a,b show the sum and difference of the mean and standard deviation per session of the PPG waves of younger healthy controls. In Fig. 3a, the newly measured PPG data from subject #6 in each session are shown as circles between the upper and lower lines. The lines in Fig. 3a,b shows the statistical data obtained from younger healthy control subjects. Figure 3b shows the differences between the statistical data of the control subjects and the values for subject #14, who suffered from hypertension. In Fig. 3c, as the PPG values of subject #14 are between the lines corresponding to the mean ± standard deviation of the elderly patients with hypertension, subject #14 was judged to belong to the patient group. However, Fig. 3d shows that the data of subject #6 are distinct from those of the patient group.

**Figure 4:**
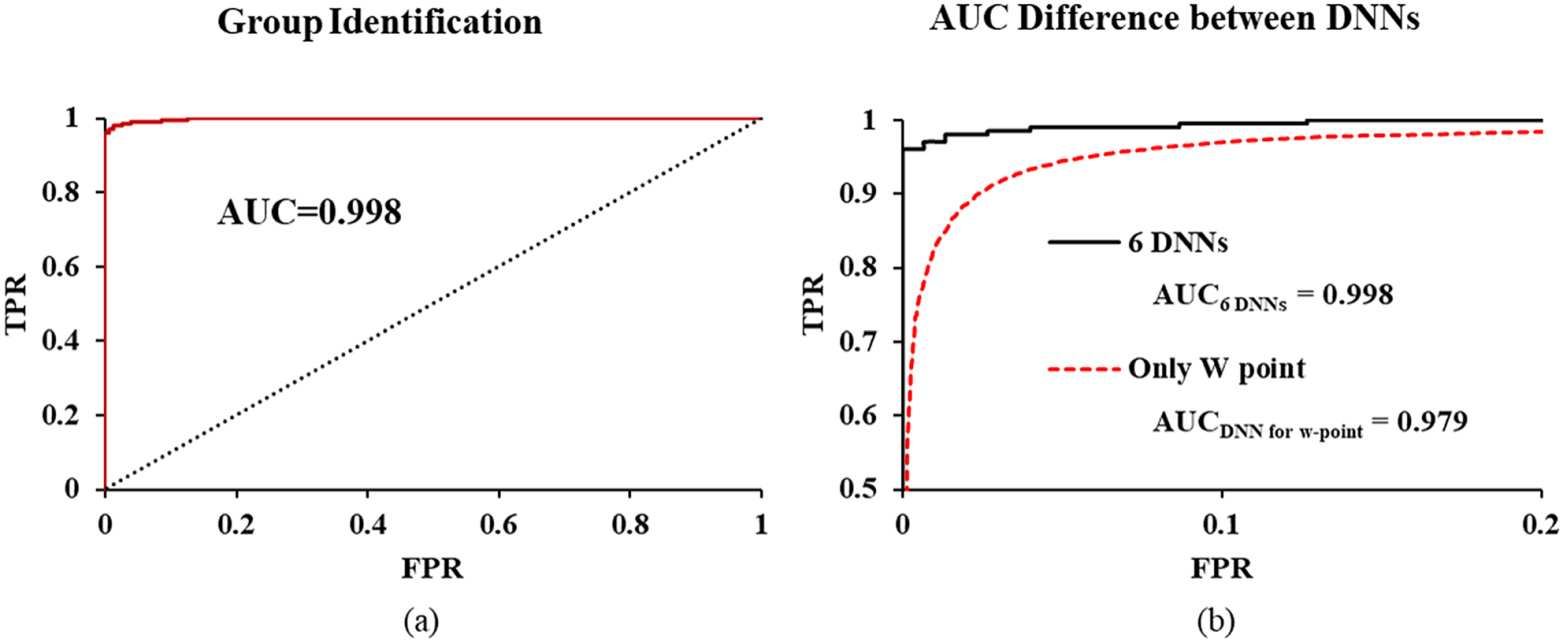
Accuracy of group identification for (**a**) reference data (75) and (**b**) test data (353).

Figure 4a shows the accuracy of the data classification (healthy control or patient group) based on analysis of the ROC curve, calculated from the difference between the measured data and the Z-values. Figure 4b shows the classification accuracy of unknown and newly obtained data for 353 dataset (healthy controls dataset, n = 150(8 subject); patients dataset, n = 203(7 subject)) not included in the model-building process. The AUC value was 0.998.

## Discussion

Previous studies used DNN techniques to analyze PPG and obtain reliable parameters related to cardiovascular disease^10,12–19^. Our team has also developed various DNN models that recognize and locate the characteristics of pulses appearing in PPG waveforms such as the peaks and staring points of the pulses caused by the heartbeat and the peaks and the starting points caused by the reflected waves and noise^22^. Finding new kinds of features in PPG waveforms required an additional machine learning process using new training data for the new DNN. However, by using the output of several previously developed neural networks rather than training a new DNN, it is possible to more reliably determine the regions of noise and to measure heartrate and heartrate variability more accurately^23^. In addition, it was possible to determine the possibility that the SPO2 monitor was erroneous from the PPG signal^24^. The already developed telemedicine management system uses 6 DNNs to select and provide reliable PPG measurement signals and other medical signals.

If the algorithm proposed in this study is applied to a remote medical information system, it is possible to increase the efficiency and reliability of medical data collection by automatically requesting the patient to remeasure the questionable data when some data differ significantly from previous data. A remote medical information system that can remove unreliable data automatically, thus reducing the amount of data that must be inspected by staff, will decrease operation costs.

As shown in Fig. 2, identification of individuals using our algorithm is not entirely reliable because of the high error rate (> 20%). It would be useful if the system could distinguish mistakenly transmitted PPG waveforms of family members coresiding with the patient. When the patient’s re-transmitted data still differ from their statistical norm, a message could be sent to the medical staff instructing them to inspect the medical data and identify the cause of the changes in the patient’s condition. Therefore, the individual identification function can contribute to the efficient management of telemedicine data.

As shown in Fig. 4, group classification accuracy was very high (AUC − 0.998), because the peaks corresponding to heartbeat and reflection were higher in the young healthy subjects in Group 1 than in the old patients with hypertension in Group 2. However, there were significant differences in both age and disease type between those groups in this study, so it is not clear which of these two factors the differences were due to. Takazawa et al. reported larger reflection waves in young people. In addition, the amplitudes of reflection waves are lower in patients with cardiovascular disease than in healthy controls of the same age^6,25^. The number of patients and datasets is insufficient to characterize the PPG features affected by diseases. Analyzing PPG by collecting more PPG data from more patients and dividing it into detailed factors such as the patient's age, gender, disease, and body size can observe how these factors affect PPG^3–8^. However, even if the disease characteristics of the patient are clearly identified, irregular changes appear among the PPGs of the same patient as shown in the standard deviation shown in Fig. 1a,d, so it may not be accurate to diagnose with the PPG waveform measured once. In addition, it is well known that temporary factors such as posture, exercise, and stress contribute to irregular changes in the PPG waveform^5–8^. Therefore, in this study, the fact that the PPG difference was clear between the groups with large differences in characteristics such as age and disease is not to show that it was useful for diagnosing the disease, but to show that, despite the large fluctuations in the PPG, the suggested DNN normalizing method could be useful for the comparison among individuals and groups.

The PPG waveform appears in a very simple form showing the pulse of heartbeat and the reflected waveforms from the renal, femoral, and iliac arteries that are overlapped or interfered. Since the number of observable pulses is less than 4, 10 segments during the heartbeat cycle are sufficient for accurate analysis on the PPG waveform. In this study, no errors were observed in the process of segmenting PPG waveform and obtaining data. However, as shown in Fig. 1, since the deviation of each segment is large from 8.8 to 21.4% of the vPPG amplitude, it is necessary to obtain the average of each segments repeatedly measured over 30 times to obtain the characteristics of each segment^26^. As shown in Figs. 1 and 3, in each segment, the average value of a target PPG were compared to 3 reference datasets. One is the previously measured individual segment values, another is the normal group's and the other is the patient group's. As a result, when once the PPG is measured, the algorithm generates 30 z values; The z value used in this study is a commonly used parameter representing the difference between the target value and the reference value using the average value and the standard deviation. The absolute z value was averaged to comprehensively determine the difference or similarity between the target and the reference. Thus, a low mean z-value indicates that the measured PPG and reference are very similar, and the high mean z-value indicates that the measured PPG and reference are very different. Since segmental z-values and mean z-values are usually obtained using the average and standard deviation of more than 30 measurements, if there is an error in setting segments using the DNN algorithm presented in this study, or if there was no differences between the patient and the normal group in each segment, personal recognition and classification of the patient group and the normal group as shown in Figs. 2 and 4 would not have been possible.

The standard deviations of each segment obtained with one DNN are larger than 6 DNNs, because the noise (less than 10% of total heartbeat) removed by 6 DNNs are included. However, the average values obtained from multiple measurements using one DNN are not significantly different from those obtained using six DNNs. Therefore, the actual accuracy of the segmentation using one DNN was not significantly affected as shown in Fig. 4b. The comparison with such one-DNN algorithm shows that the improved accuracy of the method presented in this study is due to the improvement of the accuracy of the average value by acquiring a large amount of data. However, the evaluation of the one-DNN algorithm has a limitation that the results of one-DNN algorithm were still affect by 6 DNNs, because 6 DNNs have already been applied to the management program of the telemedicine network server and removed patient data including large error. In other words, since the data determined as noise in more than 10% of heartbeats by the 6-DNNs algorithm were not saved and the participants were asked to re-measure, the error was reduced. Unsaved data accounts for 21% of all transmitted data.

The existing research method using DNN is to determine Group 1 or Group 2 directly with one DNN without a segmentation algorithm. However, in order to apply machine learning for a DNN, new training data must be created according to the classification target, and it is often difficult to create actually necessary training data. Physionet's data used as training data for 6 DNNs did not include the patient's age and disease information, so it is fundamentally difficult to create new training data for judging Group 1 and Group 2. As a results, it will be possible to determine whether the existing DNN method is more accurate than the suggested algorithm in this study in the function of distinguishing Group 1and Group 2 through a large amount of additional data in the future. In addition, if Group1 and Group2 were judged with only a DNN model, no basis for judgment would not be presented other than the judgment result. Since the proposed algorithm can show the degrees and positions of differences in PPG waveforms of other people or other group, even if a new classification criterion is created other than Group 1 or Group 2 in the future, it has the advantage that direct comparisons are possible without additional DNN models or machine learning.

## Methods

### PPG analyzing process

PPG waveform is caused by a decrease or increase in the amount of infrared light absorbed by blood vessels as arteries are dilated or contracted according to arterial blood pressure. PPG has a very similar waveform to IBP, however, PPG alone cannot be used to measure blood. Waveforms measurable in PPG are pulses caused by heartbeat and pressure pulse reflection from blood vessels^3–5^. Although Commercial SPO2 monitors first measures PPG to measure SPO2 and HR, due to errors caused by reflected waves, devices that yield more informative parameters through PPG analysis have not been developed. In commercialized SPO2 devices, the sampling frequency used for PPG measurement is 62.5 ~ 125 Hz, because the PPG waveform is a simple form in which a heartbeat pulse and 0 to 3 reflected waves are observed. In commercialized SPO2 devices, the sampling frequency used for PPG measurement is often 62.5 ~ 125 Hz, since the PPG waveform is a simple form in which a heartbeat pulse and 0 to 3 reflected waves are observed and fast sampling is not required. PPG waveform has been converted to vPPG and aPPG. The differential value of PPG, vPPG is used for normalization of PPG. The derivative of vPPG, aPPG is useful for detecting heartbeat signals, but is only used for only HR measurement in the commercial devices due to the attenuation of reflection waves and noise susceptibility. The characteristic points of PPG generally used in the previous studies that analyzed PPG are the Onset (O) point, which is the starting point of the first pulse caused by the heartbeat, and the Systolic (S) point, which is the peak point of the first pulse. The characteristic points of vPPG are the W point, which is the highest peak of first pulse and the Z point which is the first peak of reflection waves^11^. The DNN models used in this study play the role of finding the O point, S point, W point, and Z point.

The input data of the DNN models consists of PPG and vPPG waveform values which are selected in the range of 1.2 s, and 60 values which indicates the locations of the highest and lowest peaks of PPG and vPPG in the range of 0.3 s. In order to analyze the PPG measured for more than 30 s, the first input data of DNN models was selected from the data in the range of 1.2 s measured first and then processed with DNNs. By shifting the selected range of input data every 16 ms, the entire PPG waveform is continuously processed by DNNs^22,23^.

**Figure 5:**
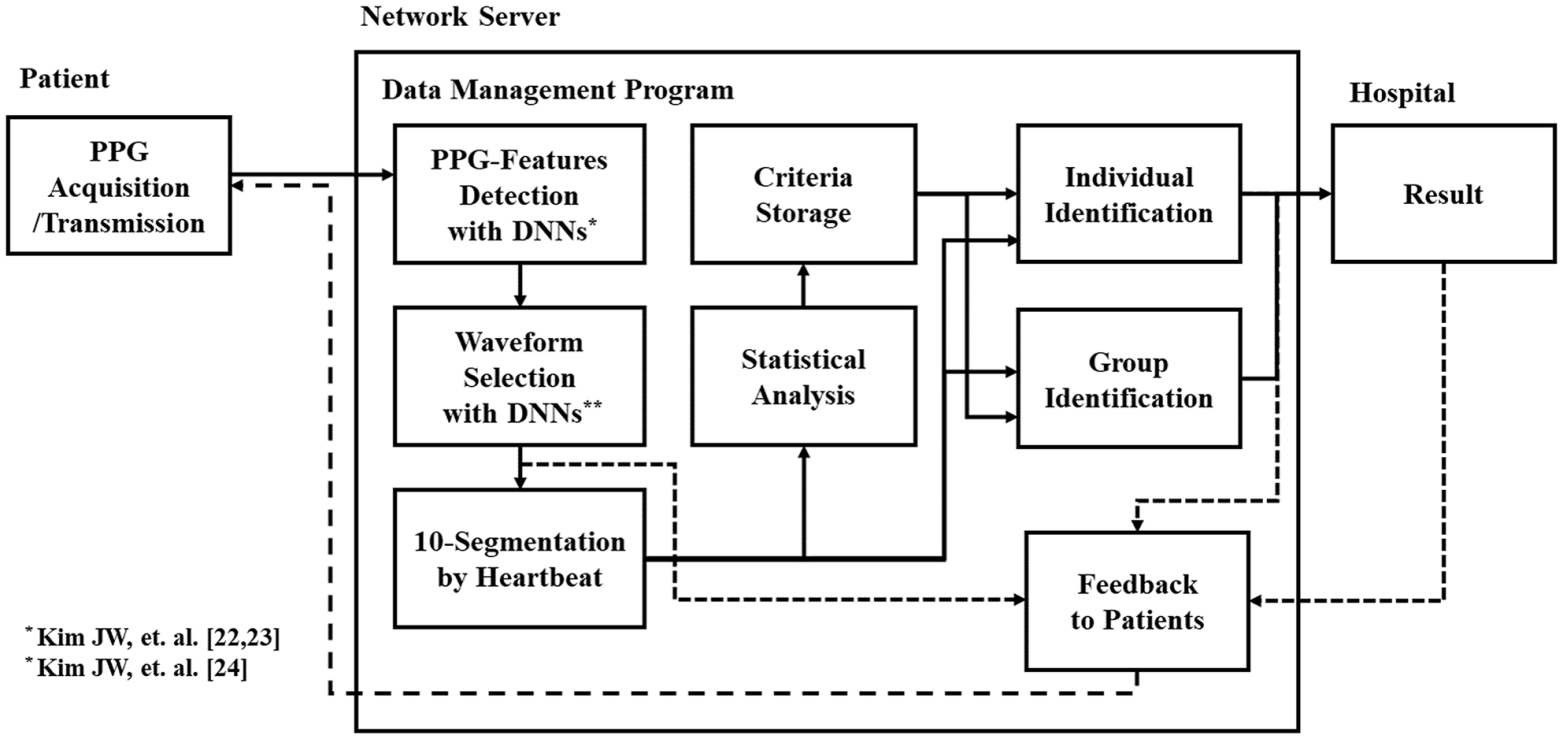
Block diagram of the PPG waveform analysis system, which incorporates DNN models for individual identification and group identification.

As shown in Fig. 5, the 6 DNNs receive the same input data at the same time to find different feature points, and the 4 DNNs find the O, S, W, Z points, which are the feature points of the heartbeat and reflection wave of the PPG and vPPG. The other 2 DNNs find the regions of sensor connection failure or motion artefact as the starting and ending points of the error^22,23^. If the rate of abnormal heart beats in the PPG exceeds 10% of the PPG measured once, a message requesting re-measurement is sent to the user^24^. Among the data transmitted to the remote server, 21% of the PPG data exceeded the error criterion, however, the finally collected 353 data include less error than 10% of entire heartbeats. The number of heartbeats in a PPG data was from 31 to 48; The heartbeats were determined by combining the results of 6 DNNs.

The 6 DNNs were trained with PPG data from 30 patients provided by Physionet (https://physionet.org) for machine learning^27,28^. The inputs data sets of the training data for 6 DNNs are all the same, and only the target data sets were modified differently for the purpose of each DNN. The learning data did not include the data of which subjects in Group 1 and Goup 2 were measured for the evaluations in this study. Each DNN has two hidden layers that are consisted of 124 neurons and 21 outputs. And, since the number of neurons in each DNN is small (269), 91 ~ 98% accuracies could be obtained with small training data from 30 people. Although the number of neurons in 6 DNNs is smaller than a conventional CNN or LSTM, the calculation speed is fast enough to allow real-time evaluations and the accuracy of heartbeat recognition could be improved by using the algorithm that verifies the outputs of 6 DNNs by comparing with each other.

**Figure 6:**
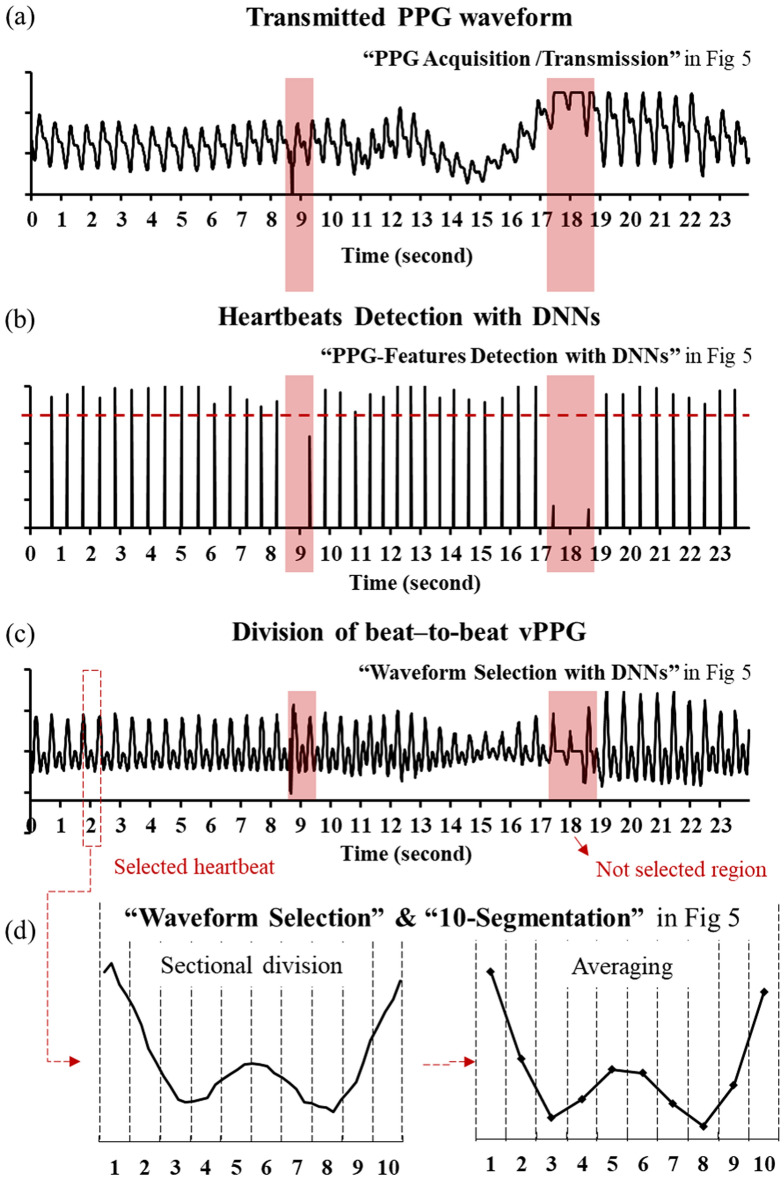
Data processing steps: (**a**) PPG data are transmitted; (**b**) Informative waveforms are selected using DNNs; (**c**) PPG waveforms are converted to the PPG velocity (vPPG) and divided for each heartbeat; and (**d**) Each waveform of vPPG is divided to 10-segment values.

Since the DNN that detected the W points was more accurate (98% accuracy) than other DNNs (91 ~ 97% accuracy), the criterion for beat-to-beat splitting was based on the W points^23^. As shown in Fig. 6, after normalizing the amplitude of beat-to-beat PPGs, those were converted to beat-to-beat vPPG by differentiating. The beat-to-beat vPPG was divided into 10 segments, and the average of each segment was obtained to determine the segment value, and the beat-to-beat vPPG was converted into 10 segment values. For 31 to 48 beat-to-beat vPPG segments in one dataset measured for 30 s, the average value and standard deviation were calculated for each segment. As a result, one dataset measured from a subject was converted into 10 segment values that are represented as mean and standard deviation. The z value was used to obtain the normalized statistical difference value by comparing two datasets expressed as the mean and standard deviation at each segment. After comparing 10 segment values against one criterion which has 10 segments values, 10 z values were determined. The mean z value, which is the average of the amounts of each z value, was obtained to comprehensively determine commonalities and differences. Since these segment values were slightly different for each subject in same group, it is necessary to evaluate whether individual identification between 15 subjects is possible.

On the other hand, it is necessary to find the commonalities of subjects by obtaining the average of each group at each segment and compare them with the segment values of other group. In this study, individual’s segment values were obtained for the criteria of individual identification using 5 datasets measured at different times from 15 subjects as shown in Table 2. Of these, 40 datasets obtained from 8 subjects were used to obtain segment values to be used as a common criteria for Group 1. In addition, with 35 datasets obtained from 7 subject, segment values were obtained for use as a common criteria for Group 2. After measuring the datasets used for the criteria, 353 section values were obtained in the same way by newly measured once a day from 15 subjects.

**Table 2:** Demographic and clinical data of all subjects.

Classify	Number	Age	Gender	Feature			Data	
				Antihypertensive medication	Systolic blood pressure	Diastolic blood pressure	Reference	Test
Group 1	#1	26	M	X	133	81	5	7
	#2	25	F	X	108	65	5	15
	#3	29	M	X	127	87	5	21
	#4	25	M	X	120	62	5	25
	#5	37	M	X	126	84	5	15
	#6	26	M	X	110	78	5	18
	#7	29	M	X	131	84	5	32
	#8	27	M	X	123	78	5	17
Group 2	#9	69	F	O	142	67	5	58
	#10	72	F	O	130	70	5	9
	#11	78	M	O	140	70	5	34
	#12	63	F	O	141	85	5	23
	#13	64	M	O	151	85	5	23
	#14	65	M	O	100	57	5	40
	#15	72	F	O	130	60	5	16

### PPG measurement

The PPGs measured from Group 2 in this study were collected from a clinical trial to evaluate the reliability of a telemedicine system with the approval of the institutional review board of Kangwon National University Hospital (KNUH-A-2020-06-008). Subjects in Group 2 were selected as patients who taking hypertension medications regularly at home, and patients with complications or hospitalization were excluded. In order to compare the patient data and evaluate the accuracy of group identification, the PPGs of young and healthy subjects were also measured according to an additional protocol (KNUH-2019-11-007) approved by the institutional review board of Kangwon National University Hospital. Written informed consent was obtained from each participant who voluntarily decided to participate after receiving an explanation of this study. All methods were carried out in accordance with relevant guidelines and regulations.

Subjects installed a sensor (Nellcor SPO2 sensor, Nellcor inc., USA) on his/her finger to measure SPO2 and PPG. When a subject measures SPO2 (PPG) using a SPO2 monitor (Pulseoximetry, Nellcor inc., USA) that are installed to a patient monitor (BPM-190, Bionics, Korea), BPM-190 converts PPG signal to digital data at 62.5 Hz sampling rate and transmits the data to a server through internet; The SPO2 monitor measures PPG with an infrared LED and a photo sensor at the first stage of SPO2 measuring process, and then, when the PPG was the highest or the lowest, the absorption rates of a red light from LED were measured to obtain SPO2 value. Most SPO2 devices and patient monitors display PPG waveform and calculate HR with PPG. However, automated analysis of PPG has not yet been applied to patient monitors or SPO2 devices.
